# *Begonia guangdongensis*, a new species of *Begonia* (Begoniaceae) from Guangdong, China

**DOI:** 10.3897/phytokeys.162.51913

**Published:** 2020-10-07

**Authors:** Wen-Hui Tu, Bing-Mou Wang, Yi Huang, Gang Yao, Jiu-Xiang Huang, Yu-Ling Li

**Affiliations:** 1 South China Limestone Plants Research Center, College of Forestry and Landscape Architecture, South China Agricultural University, Guangzhou 510642, China South China Agricultural University Guangzhou China; 2 Panyu Central Hospital, Guangzhou 511400, China Panyu Central Hospital Guangzhou China; 3 Yangchun, Guangdong, China Unaffiliated Yangjiang China

**Keywords:** *Begonia* sect. *Coelocentrum*, Guangdong, limestone karsts, new taxon

## Abstract

A new species of Begonia section Coelocentrum, *B. guangdongensis* W.H. Tu, B.M. Wang & Y.L. Li from Guangdong Province, China, is described and illustrated here. Morphologically, the new species is most similar to *B. biflora* T. C. Ku and *B. longistyla* Y. M. Shui & W. H. Chen, but differs from *B. biflora* by its rugose leaves and glabrous capsules and from *B. longistyla* by its glabrous stipules without ciliate margin, densely hirsute-pilose leaves and obtuse apex of bracts. Additionally, it is also somewhat similar to *B. chongzuoensis* Yan Liu, S. M. Ku & C.-I Peng, but there are significant distinctions in their stipules, leaves and bracts. The conservation status of *B. guangdongensis* is assessed as Critically Endangered (CR), according to the IUCN Red List Categories and Criteria.

## Introduction

The genus *Begonia* L. (Begoniaceae), consisting of ca. 1900 species, is one of the ten most species-rich flowering plant genera and is widely distributed in the tropical and subtropical areas of the world (Frodin 2004; Hughes et al. 2015). Most *Begonia* species are narrowly distributed, especially those in limestone karsts (Tebbitt et al. 2006; Ku et al. 2007; Hughes and Hollingsworth 2008). According to the recent taxonomic revision of the genus *Begonia* in Flora of China, nearly 200 species, with 141 local endemics, are reported and represent seven sections (Ku 2007; Ku et al. 2007). The Begonia sect. Coelocentrum, comprising of more than 70 species, is a typical limestone group confined to the Sino-Vietnamese karst areas and most species circumscribed within the section are rare and known only from a single collection or population (Chung et al. 2014; Peng et al. 2014). Although the section has been shown to be paraphyletic, based on phylogenetic analyses, this section is morphologically well delimited by its parietal placentation and rhizomatous perennation (Chung et al. 2014). Species within this section differ from one another by leaf texture, pubescence and stipule, inflorescence and fruit morphology (Ku et al. 2007).

During a plant diversity survey around Yangchun City in Guangdong Province in October 2019, we discovered a species of *Begonia* with parietal placentation and rhizomatous perennation on the slope of a limestone hill, which was identified as a member of Begonia sect. Coelocentrum. After critical reviewing the type specimens and protologues of relevant species of this section described from the Sino-Vietnamese karst regions, it was concluded that the species is new to science. Herein, we describe and illustrate it, as well as assess its conservation status.

## Taxonomy

### Begonia guangdongensis

Taxon classificationPlantaeCucurbitalesBegoniaceae

W.H.Tu, B.M.Wang & Y.L.Li sp. nov.

0DC1CAC1-667D-5BB6-9185-D5D17A142C36

urn:lsid:ipni.org:names:77211928-1

Figs 1 , 2

#### Diagnosis.

*Begonia guangdongensis* is morphologically similar to *B. biflora* T. C. Ku (Wu and Ku 1997), *B. longistyla* Y. M. Shui & W. H. Chen (Shui and Chen 2005) and *B. chongzuoensis* Yan Liu, S. M. Ku & C.-I Peng (Peng et al. 2012) by sharing obliquely ovate asymmetric leaves, hairy petioles, a glabrous peduncle, 2–3 times branched dichasial cyme and glabrous trigonous-ellipsoid capsules. However, it can be easily distinguished from *B. biflora* by its stipules with aristate apex and without ciliate margin (vs. aristate and ciliate apex and ciliate margin), rugose leaves (vs. flat) and glabrous capsules (vs. pubescent); it differs from *B. longistyla* by its abaxially glabrous stipules without ciliate margin (vs. abaxially hairy stipules with ciliate margin), leaves densely hirsute-pilose (vs. densely tuberculate-based pilose-setula) and inflorescence equal or slightly shorter than petioles (vs. much longer) and it can be distinguished from *B. chongzuoensis* by its persistent stipules and bracts (vs. caducous), rugose leaves (vs. nearly flat), leaves densely white hirsute-pilose (vs. moderately to sparsely whitish-hyaline or reddish setulose) and an unequally 3-winged capsule (vs. equal or subequal). (Table 1).

**Figure 1.**
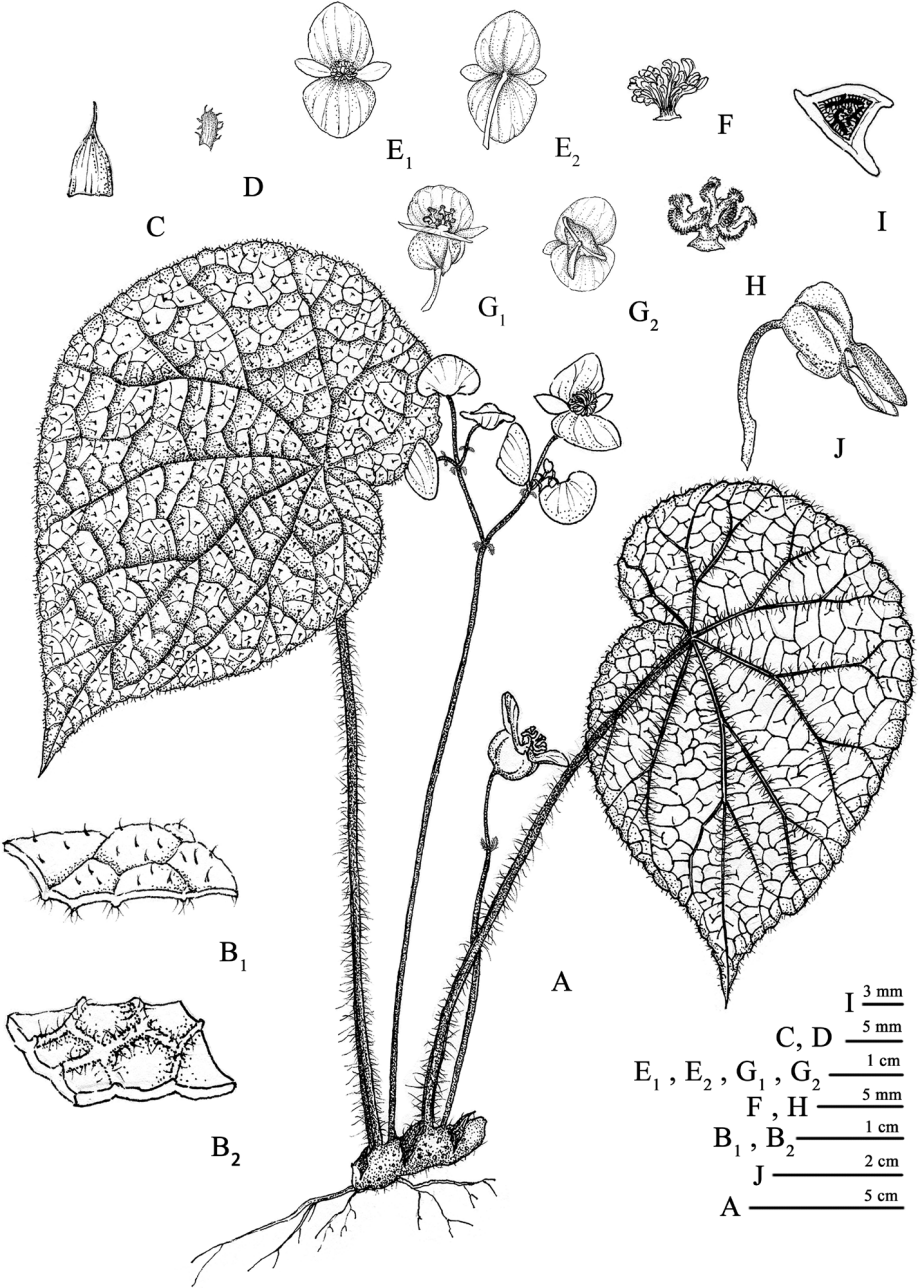
*Begonia guangdongensis***A** plant **B_1_** close up of adaxial surface of leaf **B_2_** close up of abaxial surface of leaf **C** stipule **D** bract **E_1_** and **E_2_** staminate flower **F** androecium **G_1_** and **G_2_** pistillate flower **H** style and stigma **I** cross section of ovary in the middle part **J** immature capsule. Drawn by Zheng-meng Yang.

**Table 1.** Difference between *Begonia guangdongensis*, *B. biflora*, *B. longistyla* and *B. chongzuoensis*.

Character	*B. guangdongensis*	*B. biflora*	*B. longistyla*	*B. chongzuoensis*
Stipules	persistent, ovate-triangular, apex aristate, margin eciliate, abaxially glabrous	persistent, ovate-triangular, apex aristate and ciliate, margin ciliate, abaxially glabrous or with few hairs on midrib	persistent, triangular, apex aristate, margin ciliate, abaxially with hairs	caducous, ovate or triangular-ovate, apex aristate, margin eciliate or sparsely ciliolate, abaxially glabrous or with few hairs along midrib
Petioles	ca.15–30 cm long, densely white villous	4–22 cm long, hirsute-villous	3–5 cm long, densely covered with strigae	4.5–15 cm long, sparsely hirsute-villous
Leaf blades	10–18 × 7–13 cm, apex acuminate or caudate, margin irregularly repand serrate, adaxial surface rugose, densely white hirsute-pilose, veins depressed	8–25 × 7–23 cm, apex obtuse, sometimes rounded or acute, margin crenulate and irregularly denticulate, adaxial surface flat, sparsely setulose or hispidulous, veins not depressed	6–10 × 4–6 cm, apex rotundate or with an obtuse tip, margin serrulate, adaxial surface rugose, densely tuberculate-based pilose-setulose, veins depressed	6–13 × 5–10 cm, apex acuminate or shortly acuminate, margin crenate-denticulate, adaxial surface nearly flat, moderately to sparsely whitish-hyaline or reddish setulose, veins slightly depressed
Bracts	persistent, oblong, apex obtuse	persistent, oblong or long ovate, apex undescribed	persistent, ovate, apex with a tip	caducous, ovate or rounded, apex obtuse or rounded
Inflorescence	6–15 flowers, peduncle glabrous, 15–20 cm, equal or slightly shorter than petioles	4–13 flowers, peduncle glabrous or sparsely pilose, 5–7.5 cm, shorter than petioles	20–40 flowers, peduncle glabrous, 4–8 cm long, much longer than petioles	4–8 flowers, peduncle glabrous, 5–12 cm long, shorter than petioles
Staminate flower	outer tepals 9–14 × 8–13 mm, inner tepals oblong or narrowly elliptic, 7–9 × 3–4 mm	outer tepals 4–11 × 5–9 mm, inner tepals obovate or elliptic, 6–9 × 3–5 mm	outer tepals 4–5 mm in diam., inner tepals obovate, 4.5–5 × 3–3.5 mm	outer tepals 11–14.5 × 11–15 mm, inner tepals obovate or narrowly obovate, 9–11 × 3.5–5 mm
Pistillate flower	outer tepals 6–9 × 8–12 mm, inner tepals oblong or ovate-lanceolate, styles yellow	outer tepals 6–9.5 × 6–8 mm, inner tepals oblanceolate, styles yellowish-green	outer tepals 4–5 mm in diam., inner tepals obovate, styles yellow	outer tepals 9.5–11.5 × 10–11.5 mm, inner tepals elliptic or broadly lanceolate, styles yellow
Capsules	trigonous-ellipsoid, unequally 3-winged, glabrous, with a few small red spots	oblong, unequally or subequally 3-winged, pubescent	ovate, subequally 3-winged, glabrous	trigonouse-llipsoid, somewhat compressed, equally or subequally 3-winged, glabrous
Flowering time	September to October	May	April to June	May to September

#### Type.

China. Guangdong Province, Yangchun City, Chunwan Town, on a slope of a limestone hill in an evergreen forest, 22°21'44.04"N, 111°57'26.28"E, alt. 88 m, 6 October 2019, *Li et al. 263* (holotype, CANT!; isotype, IBSC!).

#### Description.

Perennial herbs, rhizomatous. Rhizomes creeping, red, stout, 7–12 mm in diam., internodes 4–7 mm long, sparsely hairy. Leaves simple and alternate; stipules generally persistent, ovate-triangular, 5–8 × 3–5 mm, apex aristate, arista ca. 1.5 mm long, abaxially glabrous; petioles red, ca. 15–30 cm long, with densely white villose, ± reflexed trichomes; blades basifixed, asymmetric, obliquely ovate, 10–18 × 7–13 cm, papery, rugose, adaxially densely white hirsute-pilose, veins depressed, abaxially hirsute-pilose, denser on primary veins, veins convex, base obliquely deeply cordate, apex acuminate or caudate, margin irregularly repand serrate and ciliate; basal palmate veins 6–7. Inflorescences axillary, arising directly from rhizome, flowers 6–15 in a 2–3 times branched dichasial cyme; peduncles 15–20 cm long, equal or slightly shorter than petioles, glabrous; bracts oblong, 2–3 × 1–1.5 mm, apex obtuse, margin serrulate and ciliate. Staminate flowers: pedicel 1–1.5 cm long, glabrous; tepals 4, outer 2 ovate to suborbicular, 9–14 × 8–13 mm, upper side pinkish-white, lower side pink with red nerves, glabrous on both sides, inner 2 white, oblong or narrowly elliptic, 7–9 × 3–4 mm, glabrous; androecium actinomorphic, nearly spherical, yellow, stamens numerous, filaments nearly free, 1–1.5 mm long, anthers obovate, ca. 1.2 × 0.7 mm, apex emarginated. Pistillate flower: pedicle 1–1.5 cm; tepals 3, outer 2 broadly ovate to suborbicular, 6–9 × 8–12 mm, pink with red nerves, glabrous on both sides, inner 1 of left side white, oblong or ovate-lanceolate, 5–6 × 2–3 mm; styles 3, fused at base, yellow, ca. 1.5–2 mm long, the upper 2-cleft; stigmas spirally twisted; ovary trigonous-ellipsoid, dark pink, 1-locular with parietal placentation, glabrous, 3-winged. Capsule nodding, trigonous-ellipsoid, apex obtuse, 8–10 mm long, 5–7 mm in diam. (wings excluded), surface with a few small red spots, unequally 3-winged, abaxial wing lunate, 2.5–5 mm wide, lateral wings 2–3 mm wide, glabrous.

**Figure 2.**
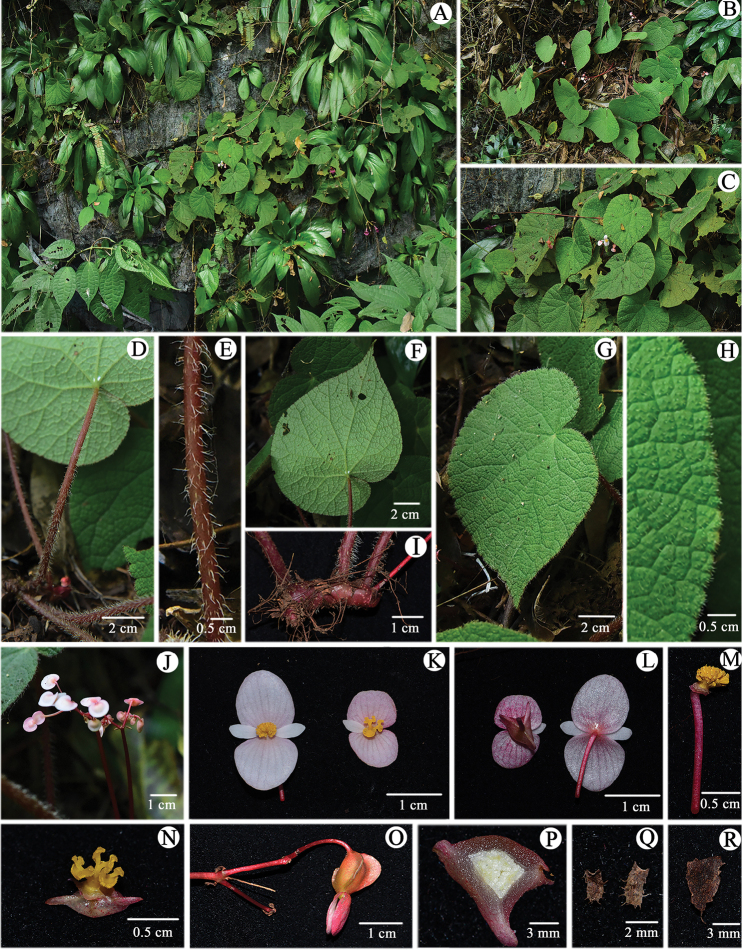
Habitat and morphology of *Begonia guangdongensis***A, B** habitat **C** habit **D** petiole **E** close up of petiole **F** view of abaxial surface of leaf **G** view of adaxial surface of leaf **H** close up of adaxial surface of leaf **I** rhizome **J** inflorescences **K** view of adaxial surfaces of staminate and pistillate flower **L** view of abaxial surfaces of staminate and pistillate flower **M** androecium **N** styles and stigmas **O** immature capsule **P** cross section of ovary in the middle part **Q** dry bract **R** dry stipule.

#### Phenology.

Flowering in September to October, fruiting in October to November.

#### Etymology.

The new species is named after the type locality, Guangdong Province, China.

#### Habitat.

**Figure 3.**
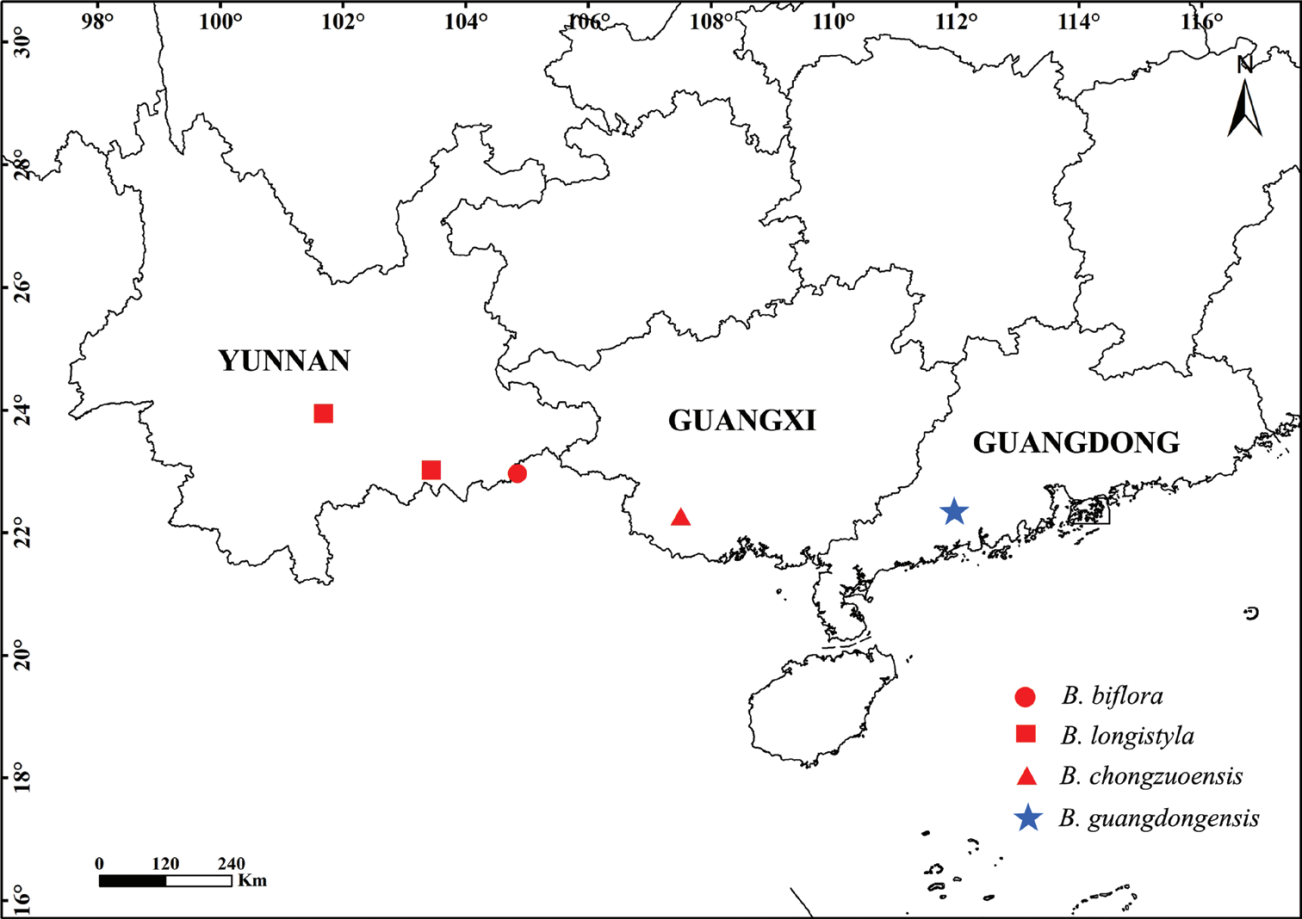
Distribution map of *B. biflora*, *B. longistyla*, *B. chongzuoensis* and *B. guangdongensis*.

This new species grows on the slope of a limestone hill in evergreen forests at an elevation of 80–100 m (Fig. 3).

#### Distribution.

Only one population of this new species was discovered in Guangdong Province in China.

#### Conservation Status.

**Critically Endangered (CR)**. Limestone areas in Chunwan Town have been searched for this new species, but *Begonia guangdongensis* is known only from one population consisting of ca. 100 mature individuals. The area of occupancy (AOO) of the species is estimated to be less than 4 km^2^, which indicates the species belongs in the Critically Endangered category under criterion B2, according to the IUCN Red List Categories and Criteria (IUCN 2019). Since the species grows on a limestone hill near two cement factories, the species is threatened by the limestone quarrying. Its habitat will likely be destroyed since the area is undergoing a continuing decline. Based on the current information (one location with area in continuing decline and AOO less than 10 km^2^), the new species can be assessed as Critically Endangered [B2ab(iii)] (IUCN 2019).

#### Discussion.

*Begonia guangdongensis*, belonging to Begonia sect. Coelocentrum, is a very distinctive species in having leaf features, such as rugose and densely hirsute-pilose leaves and an obtuse apex of the capsules. Although it is more or less similar to *B. biflora*, *B. longistyla* and *B. chongzuoensis* in their obliquely ovate asymmetric leaves and glabrous trigonous-ellipsoid capsules, it differs from *B. biflora* by its sparsely hairy and smooth rhizomes (vs. rough rhizomes with many membranous scales), leaves with densely hirsute-pilose and depressed veins on adaxial surface (vs. with sparsely setula and veins not depressed) and stipule and capsule features discussed in the above diagnostic description. However, *B. guangdongensis* is quite different from *B. longistyla*, being distinguished by its oblong bracts with obtuse apex (vs. ovate bracts with a tip apex), stipule and leaf pubescence, length of inflorescence and capsules features. *B. guangdongensis* is also markedly distinct from *B. chongzuoensis* by its stipules, leaf and bract and capsule features. In addition, their distribution range is different (Fig. 3). Both *B. biflora* and *B. longistyla* are distributed in Yunnan Province and *B. chongzuoensis* in Guangxi Province, whereas *B. guangdongensis* occurs in Guangdong Province. Additionally, *B. guangdongensis* flowers in September to October, while *B. biflora* flowers in May, *B. longistyla* in April to June and *B. chongzuoensis* in May to September. Thus, even if they were growing together, they would be genetically isolated in time.

## Supplementary Material

XML Treatment for Begonia guangdongensis
